# Noninvasive assessment and therapeutic monitoring of drug-resistant colorectal cancer by MR molecular imaging of extradomain-B fibronectin

**DOI:** 10.7150/thno.47448

**Published:** 2020-09-08

**Authors:** Amita Vaidya, Nadia Ayat, Megan Buford, Helen Wang, Aman Shankardass, Yiqing Zhao, Hannah Gilmore, Zhenghe Wang, Zheng-Rong Lu

**Affiliations:** 1 Department of Biomedical Engineering, Case Western Reserve University, Cleveland, OH 44106, USA.; 2 Department of Genetics and Genome Sciences, School of Medicine, Case Western Reserve University, Cleveland, OH 44106, USA.; 3 Department of Pathology, University Hospitals of Cleveland, Cleveland, OH 44106, USA.; 4 Case Comprehensive Cancer Center, Case Western Reserve University, Cleveland, OH 44106, USA.

**Keywords:** drug resistance, colorectal cancer, MRMI, therapeutic monitoring, EDB-FN.

## Abstract

Antineoplastic resistance represents a multifaceted challenge for cancer therapy and diagnostics. Extensive molecular heterogeneity, even within neoplasms of the same type, can elicit distinct outcomes of administering therapeutic pressures, frequently leading to the development of drug-resistant populations. Improved success of oncotherapies merits the exploration of precise molecular imaging technologies that can detect not only anatomical but also molecular changes in tumors and their microenvironment, early on in the treatment regimen. To this end, we developed magnetic resonance molecular imaging (MRMI) strategies to target the extracellular matrix oncoprotein, extradomain-B fibronectin (EDB-FN), for non-invasive assessment and therapeutic monitoring of drug-resistant colorectal cancer (CRC).

**Methods:** Two drug-resistant CRC lines generated from parent DLD-1 and RKO cells by long-term treatment with 5ʹ-FU and 5ʹ-FU plus CB-839 respectively, were characterized for functional and gene expression changes using 3D culture, transwell invasion, qRT-PCR, and western blot assays. Contrast-enhanced MRMI of EDB-FN was performed in athymic nu/nu mice bearing subcutaneous tumor xenografts with 40 µmol/kg dose of macrocyclic ZD2-targeted contrast agent MT218 [ZD2-N_3_-Gd (HP-DO3A)] on a 3T MRS 3000 scanner. Immunohistochemistry was conducted on patient specimens and xenografts using anti-EDB-FN antibody G4.

**Results:** Analyses of TCGA and GTEx databases revealed poor prognosis of colon cancer patients with higher levels of EDB-FN. Similarly, immunohistochemical staining of patient specimens showed increased EDB-FN expression in primary colon adenocarcinoma and hepatic metastases, but none in normal adjacent tissues. Drug-resistant DLD1-DR and RKO-DR cells were also found to demonstrate enhanced invasive potential and significantly elevated EDB-FN expression over their parent counterparts. MRMI of EDB-FN with 40 µmol/kg dose of MT218 (60% lower than the clinical dose) resulted in robust signal enhancement in the drug-resistant CRC xenografts with 84-120% increase in their contrast-to-noise ratios (CNRs) over the non-resistant counterparts. The feasibility of non-invasive therapeutic monitoring using MRMI of EDB-FN was also evaluated in drug-resistant DLD1-DR tumors treated with a pan-AKT inhibitor MK2206-HCl. The treated drug-resistant tumors failed to respond to therapy, which was accurately detected by MRMI with MT218, demonstrating higher signal enhancement and increased CNRs in the 4-week follow-up scans over the pre-treatment scans.

**Conclusions:** EDB-FN is a promising molecular marker for assessing drug resistance. MRMI of EDB-FN with MT218 at a significantly reduced dose can facilitate effective non-invasive assessment and treatment response monitoring of drug-resistant CRC, highlighting its translational potential for active surveillance and management of CRC and other malignancies.

## Introduction

Colorectal cancer (CRC) is the second most common cause of cancer death in men and women combined in the US 1. The 5-year survival for CRC cases in 2013 decreased from 88.1% at stage I to 12.6% at stage IV of diagnosis 2. Multiple studies and clinical trials have proven significantly reduced CRC mortality from preventive screening 3, 4. Although highly treatable with chemotherapy and surgery in early stages, 80-90% metastatic CRC is inoperable at diagnosis and requires neoadjuvant chemotherapy 5, 6. Furthermore, development of multidrug resistance can also lead to relapse and distant metastases, which are incurable. Current standards for CRC treatment monitoring follow the RECIST (Response Evaluation Criteria in Solid Tumors) guidelines 7, which are limited due to their reliance on tumor anatomical size, lesion irregularities leading to subjective opinions, and delays in detection of negative tumor response to therapies 8, 9. To improve the outcomes of chemo- or targeted therapies and to enable decision making for adaptive interventions, non-invasive and repeated imaging for accurate detection and surveillance of invasive, drug-resistant tumor populations is imperative.

Clinical CRC diagnostics utilize blood markers like carcinoembryonic antigen (CEA) and guaiac-based fecal occult blood test (gFOBT), which suffer from low sensitivity and specificity 10. Liquid biopsies that measure mutational burden from circulating tumor DNA (ctDNA) are reflective of inter-patient and inter- and intra-tumor heterogeneity 11, and are fast gaining momentum in the diagnostic and prognostic arenas 12; however, they cannot provide spatial information on neoplastic lesions, rendering diagnostic imaging indispensable. A common imaging modality for CRC treatment monitoring is positron emission tomography-computed tomography (PET-CT), usually with the [^18^F]-fluoro-2-deoxyglucose (^18^F-FDG) radiotracer, which provides functional and metabolic data, but is limited by radiation exposure and confounding factors like cell density, hyperglycemia, and poor resolution 5, 13, 14. Contrast-enhanced magnetic resonance imaging (MRI) employs Gd(III)-based contrast agents (GBCAs) that shorten T_1_ of tissues, and T_1_-weighted imaging is also routinely used for diagnosis and surveillance of CRC 15-18. This current clinical assessment is fraught with serious challenges in that, clinical non-targeted GBCAs are non-specific and unable to distinguish aggressive drug-resistant tumor species from the sensitive ones; and repeated administration of GBCAs is associated with potential safety concerns of Gd-based toxicity and brain deposition 19-21. Given the extensive molecular and phenotypic plasticity of CRC tumors 22, it is essential to develop molecular imaging agents and strategies that can accurately identify the trajectory of CRC during oncotherapy, based on alterations in key biologically relevant microenvironmental characteristics of individual tumors and tumor niches.

To date, the discipline of molecular cancer imaging has witnessed a limited number of studies, possibly due to the dearth of reliable biomarkers. Although colon cancer-secreted protein 2 (CCSP-2) was used as a molecular marker for near-infrared fluorescence imaging of primary tumors, patient-derived xenografts, and liver metastases, this marker is only specific to colon adenomas 23. Among the widely reported magnetic nanoparticle (MNP) studies 24, MRI-based detection of iron oxide nanoparticles targeting human tumor antigen underglycosylated mucin 1 (uMUC1) 25, and nanoaggregation of probe scaffolds specific to pro-apoptotic caspases 26, has been used to monitor chemotherapeutic response of colon xenografts. Vascular volume fraction (VVF), K-Ras mutation, EGFR, and α_v_β_3_ have been used as surrogate markers for MRI of CRC with some success 27-29. Novel theranostic approaches, combining MRI, DCE-MRI and diverse therapies like photodynamic therapy and MXene-based nanoplatforms, have also been reported for other models of cancer 30-32. However, MR molecular imaging (MRMI) for non-invasive assessment and treatment monitoring of drug-resistant CRC has never been performed before. To address this challenge, to circumvent the immune and translational hurdles of MNPs, and to potentially target a broader range of drug-resistant cancers, we developed MRMI strategies to target the abundantly expressed oncoprotein extradomain-B fibronectin (EDB-FN) in the tumor extracellular matrix (ECM) 33-35.

EDB-FN, an oncofetal isoform of fibronectin 36, is overexpressed in a multitude of cancers 37-41. EDB-FN is also elevated in CRC and is associated with angiogenesis, growth, and tissue remodeling 42, 43. Literature studies show that even within the same cancer type, EDB-FN is preferentially upregulated in the more invasive cell and tumor subtypes, compared to the indolent ones 40, 44, 45. This specific spatial and temporal expression of EDB-FN in malignancies and its absence in healthy tissues renders it an attractive target for molecular imaging and targeted therapy 46-50. We previously designed and developed an EDB-FN-targeted GBCA, ZD2-N_3_-Gd(HP-DO3A) (MT218), by conjugating the EDB-FN-specific peptide ZD2 to the clinical GBCA Gadoteridol 51, 52, and demonstrated its ability to facilitate efficient MRMI for risk-stratification of EDB-FN-rich prostate and breast cancers, even at doses as low as 20 µmol/kg (1/5^th^ of the clinical dose), highlighting its translational applications and superior safety profile 51, 53. Despite these and other EDB-FN-related studies by independent groups, the expression profiles of EDB-FN in drug-resistant CRC have never been examined. In addition, the feasibility of using EDB-FN as a molecular marker for assessing therapeutic response of drug-resistant CRC remains unexplored.

Here, we demonstrate, for the first time, that MRMI of EDB-FN with MT218 can facilitate efficient non-invasive assessment and treatment response monitoring of drug-resistant CRC tumors that exhibit significantly elevated EDB-FN levels. Subcutaneously implanted drug-resistant CRC xenografts showed robust signal enhancement with 40 µmol/kg dose of MT218, compared to their respective drug-sensitive counterparts. MRMI of EDB-FN was also used to successfully monitor the negative response of drug-resistant CRC tumors to the targeted pan-AKT inhibitor, MK2206-HCl 54, indicating the potential of EDB-FN as a therapy-predictive marker. Moreover, elevated EDB-FN correlated with poor prognosis of colon cancer patients. To date, this is the first and only report providing compelling *in vivo* evidence for exploiting drug resistance-mediated upregulation of EDB-FN as a molecular marker for imaging and therapeutic monitoring of CRC.

## Methods

### Cell culture

CRC cell lines DLD-1 and RKO were purchased from ATCC (Manassas, VA). Their respective drug-resistant derivatives, DLD1-DR and RKO-DR were a kind gift from the lab of Zhenghe Wang (CWRU, Cleveland, OH). DLD1-DR cells were developed with resistance to 5ʹ-fluorouracil (5ʹ-FU) (Millipore-Sigma, St. Louis, MO), to an IC_50_ of 210.6 µM vs IC_50_ of 2.5 µM for DLD-1 cells, as previously described 55. RKO-DR cells were developed with resistance to combined treatment of 10 µM 5ʹ-FU and 15 µM CB-839, a glutaminase inhibitor (Selleck Chemicals, Houston, TX), as shown in **Figure S1A**. The DLD-1 and DLD1-DR cells were cultured in McCoy's 5A medium (Thermo Fisher Scientific, Waltham, MA). The RKO and RKO-DR cells were cultured in RPMI-1640 medium (Millipore-Sigma). Both the media were supplemented with 10% fetal bovine serum and 100 Units/mL Penicillin/Streptomycin. All the cells were grown at 37 °C and 5% CO_2_.

### Western blot

Total protein extraction was performed by treating CRC cell pellets with cell lysis buffer (1:1 mix of protease inhibitor in PBS and Laemmli buffer), followed by incubation at 100 °C for 10 min and then centrifugation at 15,000 rpm for 15 min at 4 °C. Protein concentration of the extracts was determined using a Lowry assay kit, according to manufacturer's instructions (Bio-Rad, Hercules, CA). Equal amount of protein extracts (40 µg) was loaded on to SDS-PAGE for electrophoresis and transferred onto nitrocellulose membranes. The following primary antibodies (1:1000 dilution, overnight incubation at 4 °C) were used: anti-MDR1, anti-E-cadherin and, anti-β-Actin (Cell Signaling Technology, Danvers, MA) and anti-N-cadherin (1:500 dilution, Abcam, Cambridge, MA). Following secondary antibody incubation (1:2000 dilution for 2 h), the membranes were developed using Signal Fire Plus ECL Kit (Cell Signaling Technology, Danvers, MA) and imaged on ChemiDoc™ XRS+ Imager (Bio-Rad). The band intensities were quantified using the softwares FIJI (FIJI is Just ImageJ) and ImageLab (Bio-Rad).

### qRT-PCR

Total RNA extraction from the CRC cells was performed using the RNeasy Plus Mini Kit (Qiagen, Germantown, MD), according to manufacturer's protocol. cDNA was generated by reverse transcription with the miScript II RT Kit (Qiagen) and qPCR was performed using SyBr Green PCR Master Mix (Thermo Fisher Scientific). Relative gene expression was measured by the 2^-ΔΔCt^ method. β-Actin was used as the housekeeping gene. The following primer sequences (IDT, Coralville, IA) were used- EDB-FN: Fwd 5ʹ-CCG CTA AAC TCT TCC ACC ATT A-3ʹ and Rev 5ʹ-AGC CCT GTG ACT GTG TAG TA-3ʹ; FN1: Fwd 5ʹ-CCT GGA GTA CAA TGT CAG TG-3ʹ and Rev 5ʹ-GGT GGA GCC CAG GTG ACA-3ʹ; β-Actin: Fwd 5ʹ-CAT CCA CGA AAC TAC CTT CAA CTC C-3ʹ and Rev 5ʹ-GAG CCG CCG ATC CAC ACG-3ʹ; MDR1: Fwd 5ʹ-TTG CTG CTT ACA TTC AGG TTT CA-3ʹ and Rev 5ʹ-AGC CTA TCT CCT GTC GCA TTA-3ʹ; E-cad: Fwd 5ʹ-GTC AGT TCA GAC TCC AGC CC-3ʹ and Rev 5ʹ-AAA TTC ACT CTG CCC AGG ACG -3ʹ; and N-cad: Fwd 5ʹ-CAC TGC TCA GGA CCC AGA T-3ʹ and Rev 5ʹ-TAA GCC GAG TGA TGG TCC-3ʹ.

### Invasion and migration assays

Standard transwell assays were performed to assess the migration and invasion of CRC cells. To test migration, 100,000 CRC cells (starved overnight) were plated in transwell inserts (VWR, Radnor, PA) placed in a 24-well plate containing FBS-rich media. The next day, the inserts were internally swabbed to remove the non-migrated cells. The migrated cells at the bottom of the inserts were fixed with 10% formalin (10 min) and then stained with 0.1% crystal violet (20 min). Excess stain was washed and the transwells were dried overnight before imaging on the Moticam T2 camera with 10X objective lens. To test invasion, the transwell inserts were coated with 1 mg/mL Corning^TM^ Matrigel^TM^ Membrane Matrix (Corning, NY), to assess the ability of the CRC cells to invade through the Matrigel layer, in addition to the porous membrane of the inserts. Approximately 200,000 CRC cells (starved overnight) were plated for this assay and processed as mentioned above. The invading and migration cells were quantified using FIJI software.

### Matrigel 3D culture and ZD2-Cy5.5 staining

The ability of CRC cells to grow in 3D was tested using Matrigel culture. About 900,000 CRC cells were plated in 4-well microslides (Ibidi, Fitchburg, WI) coated with a thick layer of Corning^TM^ Matrigel^TM^ Membrane Matrix. Tumor spheroid/organoid formation was monitored and photographed for up to 4 days using the Moticam T2 camera with 10X objective lens. To test EDB-FN expression, the tumor spheroids were incubated with 100 nM ZD2-Cy5.5 and 5 µg/mL Hoechst-33342 for 20-30 min. Excess dyes were washed thrice with PBS and fluorescence imaging was performed on Olympus FV1000 confocal microscope (Japan), with 10X and 20X objective lenses. Image processing was done using FIJI.

### Mouse models

Nude athymic mice (6-week-old nu/nu females) were purchased from The Jackson Laboratory (Bar Harbor, MA) and housed in the Animal Facility at CWRU. All the animal experiments were performed according to the protocol approved by the IACUC of CWRU. For assessment of drug resistance, 2 drug-resistant and 2 non-drug-resistant models were set up. About 3-4 x 10^6^ DLD-1, RKO, DLD1-DR, and RKO-DR cells suspended in Matrigel-PBS mixture (1:1) were subcutaneously injected in the left flanks of nude mice (100 µL per mouse, 5 mice per group x 4 models = 20 mice). After 9 days when the tumors reached 100-200 mm^3^, MRMI was performed on the 4 xenograft models with 40 µmol/kg dose of MT218. The animals were then euthanized, and the tumors were harvested for post-mortem histology and immunohistochemistry (IHC).

For therapeutic monitoring, 3-4 x 10^6^ DLD1-DR cells suspended in Matrigel-PBS mixture (1:1) were subcutaneously injected in the left flanks of 10 nude mice (100 µL per mouse, mice labeled TM1-TM10). Tumor volumes were monitored and measured once a week using a Vernier caliper. When the average tumor volumes reached 100 mm^3^, mice were randomized into 2 groups of n=5: vehicle (mice# TM1, TM3, TM4, TM5, & TM10) and treated (mice# TM2, TM6, TM7, TM8, & TM9). Once a week, mice in the treated group received MK2206-HCl (100 mg/kg) and those in the vehicle group were injected with equivalent volume of DMSO, as described previously 56. After 3 weeks of treatment, tumor volumes increased over 1000 mm^3^ and the experiment was terminated. The animals were then euthanized, and the tumors were harvested for post-mortem histology and IHC. Tumor volumes were calculated as [(Width)2 x Length]/2.

### MRMI of EDB-FN with MT218

MT218 [ZD2-N_3_-Gd(HP-DO3A)] was synthesized as previously described 51. Briefly, click reaction between alkynyl-ZD2 and N_3_-Gd(HP-DO3A) was performed in the presence of CuSO_4_ and ascorbate at room temperature, followed by FLASH chromatography purification and validation of MT218 by MALDI-TOF mass spectrometry (m.w. 1443). For assessment of drug resistance and therapeutic monitoring, MRMI was performed in a 3T MRS 3000 scanner (MR Solutions, Surrey, UK) with a mouse short quad coil. The mice were anesthetized with isofluorane and tail vein catheter was setup. T_1_-weighted MR images were obtained before (pre-contrast) and 25 min after injection (post-contrast) of 40 µmol/kg dose of MT218. The following two sequences were used with respiratory gating: axial fast spin echo (FSE) (T_R_ = 305 ms, T_E_ = 11 ms, FA = 90°, FOV = 40 mm x 40 mm, slice thickness = 1 mm, slice number = 15, N_av_ = 2, matrix = 256 x 256) and coronal FSE (T_R_ = 305 ms, T_E_ = 11 ms, FA = 90°, FOV = 90 mm x 90 mm, slice thickness = 1 mm, slice number = 20, N_av_ = 1, matrix = 248 x 512). For therapeutic monitoring, baseline MRMI (week 1) and endpoint MRMI (week 4) were performed using the aforementioned sequences with 40 µmol/kg dose of MT218 on the 10 mice bearing DLD1-DR tumors. Contrast-to-noise ratios (CNRs) were calculated as (mean tumor intensity - mean muscle intensity)/standard deviation of noise. Image and CNR analysis was performed using FIJI software. ROIs were drawn around whole tumors, 2-4 muscle regions, and background. CNR analysis was performed independently by 2 individuals, once blinded, to avoid bias.

### Immunohistochemistry in human and mouse tissues

De-identified and de-classified human tissue samples, including primary colon adenocarcinoma (n=6), liver metastasis (n=4), and their corresponding normal adjacent tissues, (n=6+4), were acquired from the Human Tissue Procurement Facility at CWRU. Dissected mouse tumor tissues were fixed in 10% neutral buffered formalin, embedded in paraffin, and sectioned into 1 µm slices. Staining and IHC services were provided by the Tissue Resources Core Facility of the Case Comprehensive Cancer Center and University Hospitals of Cleveland. The slides were stained with H&E to visualize morphology. IHC was performed using anti-EDB-FN antibody G4 clone (1:100 dilution; Absolute Antibody, UK). All the slides were reviewed by a certified pathologist. IHC images were obtained using Bx61VS slide scanner microscope (Olympus) with 40X objective lens and processed in OlyVIA software.

### Gene data and statistical analyses

Kaplan-Meier curves for overall survival (OS) and disease-free survival (RFS) data for correlation with EDB-FN expression (transcript ID: ENST00000432072.6) were derived in GEPIA2 57. This web server [http://gepia2.cancer-pku.cn/#general] evaluates tumor/normal data and normal tissue data (transcript per million) from TCGA and GTEx databases, respectively, and employs Log-rank or Mantel-Cox test, for statistical survival analysis and Cox PH Model for hazards ratio (HR) calculation.

All the experiments were independently replicated at least 3 times (n = 3), unless otherwise stated. Data are represented as mean ± sem. Statistical analysis was performed using GraphPad Prism version 7.03. Data between two groups (normal distribution) was compared using unpaired *t*-test. Otherwise, non-parametric test (Mann-Whitney U test) was used, as stated in the relevant figure legends. Time course MRMI data for multiple cell lines was analyzed by 2-way analysis of variance (ANOVA) with Tukey's correction. *p*<0.05 was considered to be statistically significant.

## Results

### Acquired drug resistance enhances migration and invasion of CRC cells

**Figure 1:**
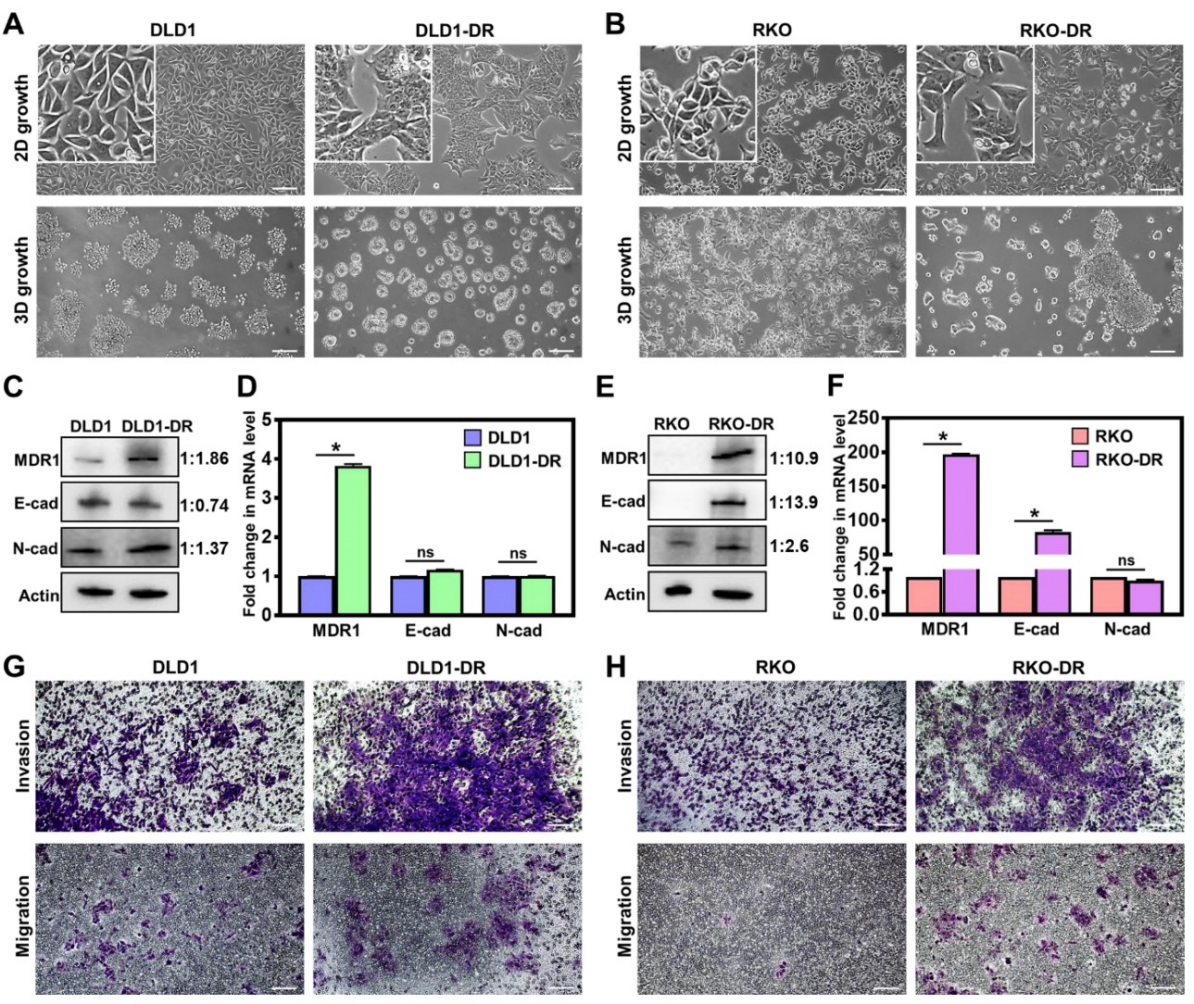
** Acquired drug resistance enhances migration and invasion of CRC cells. A.** Morphological changes in 2D monolayer and 3D tumor spheroid cultures in DLD1-DR cells with acquired resistance to 5ʹ-FU by parent DLD1 cells. **B.** Altered morphology in 2D monolayer and 3D tumor spheroid cultures in RKO-DR cells with acquired resistance to 5ʹ-FU and CB-839 by parent RKO cells. Increased expression of drug resistance marker protein MDR1 and altered levels of EMT markers N-cad and E-cad in **C, D.** DLD1-DR and **E, F.** RKO-DR cells at protein and mRNA levels using western blotting and qRT-PCR, respectively. The western blot band intensities were normalized to the β-actin control, and the protein level changes were expressed as ratio of sensitive to resistant, next to the respective lanes. Increased invasion through Matrigel layer and migration measured using transwell assay in drug-resistant **G.** DLD1-DR and **H.** RKO-DR cells, compared to their parent counterparts. Cells were stained using 0.1% crystal violet. Bars indicate mean ± sem. *p<0.05 using unpaired *t*-test. Scale bar = 100 µm.

Two independent drug-resistant CRC cell lines were generated: DLD1-DR by long-term 5ʹ-FU treatment in DLD-1 cells and RKO-DR by combined treatment of 5ʹ-FU and CB-839 in RKO-DR cells, and evaluated for their biological properties. The morphology of cells grown in 2D and 3D cultures was monitored by phase contrast microscopy. While DLD-1 cells showed regular epithelial morphology in 2D culture and multicellular grape-like clusters in 3D culture** (Figure 1A)**, DLD1-DR cells formed islets in 2D culture and compact spheroids in 3D culture. Monolayer cultures of RKO and RKO-DR cells showed no overt morphological differences **(Figure 1B)**. On the other hand, in 3D culture, RKO-DR cells readily formed tumor spheroids, both compact and aggregates, while RKO cells were unable to form spheroids. These results demonstrate the diverse morphological variation, both in monolayer and 3D culture, between the CRC cell lines.

Next, drug resistance-induced molecular changes in the signaling pathways of the CRC cells were analyzed by western blotting and qRT-PCR. Since drug efflux pumps and epithelial-mesenchymal transition (EMT) are implicated in the development of drug resistance 58, the protein and mRNA expression of multidrug resistance protein 1 (MDR1), and EMT markers (E-cadherin and N-cadherin) were determined. At the protein level, DLD1-DR cells showed significant upregulation of MDR-1 and moderate EMT, with modest decrease in E-cad and increase in N-cad levels, over DLD-1 cells **(Figure 1C)**. At the mRNA level, the DLD1-DR cells showed 4-fold increase in MDR-1, and no changes in mRNA levels of E-cad and N-cad **(Figure 1D)**. On the other hand, RKO-DR cells showed significantly elevated MDR1 and E-cad at both protein** (Figure 1E)** and mRNA **(Figure 1F)** levels, over RKO cells. The N-cad protein expression was also elevated in RKO-DR cells, suggesting the existence of a mix of epithelial and mesenchymal cells or a partial/hybrid E-M phenotype in RKO-DR cells.

The functional effects of acquired drug resistance in the CRC cells were evaluated by testing their migratory potential. As shown in **Figure 1G-H and Figure S1B-C**, DLD1-DR and RKO-DR cells exhibited increased migration through transwell membranes as well as increased invasion through an additional layer of Matrigel coated on the transwell membranes. Taken together, these results suggest that irrespective of the distinct morphological and molecular changes caused by acquisition of drug resistance, CRC cells gain significant invasive advantages over their parent counterparts.

### Acquired drug resistance is associated with elevated EDB-FN expression in CRC cells

We previously showed that EDB-FN is overexpressed in drug-resistant, invasive breast cancer 40, 45 and aggressive prostate cancer 39, 51. Here, we evaluated whether development of drug resistance upregulates EDB-FN expression in the CRC cells, using qRT-PCR and EDB-FN-specific peptide probe ZD2-Cy5.5 52 in 2D and 3D cultures, respectively.

**Figure 2:**
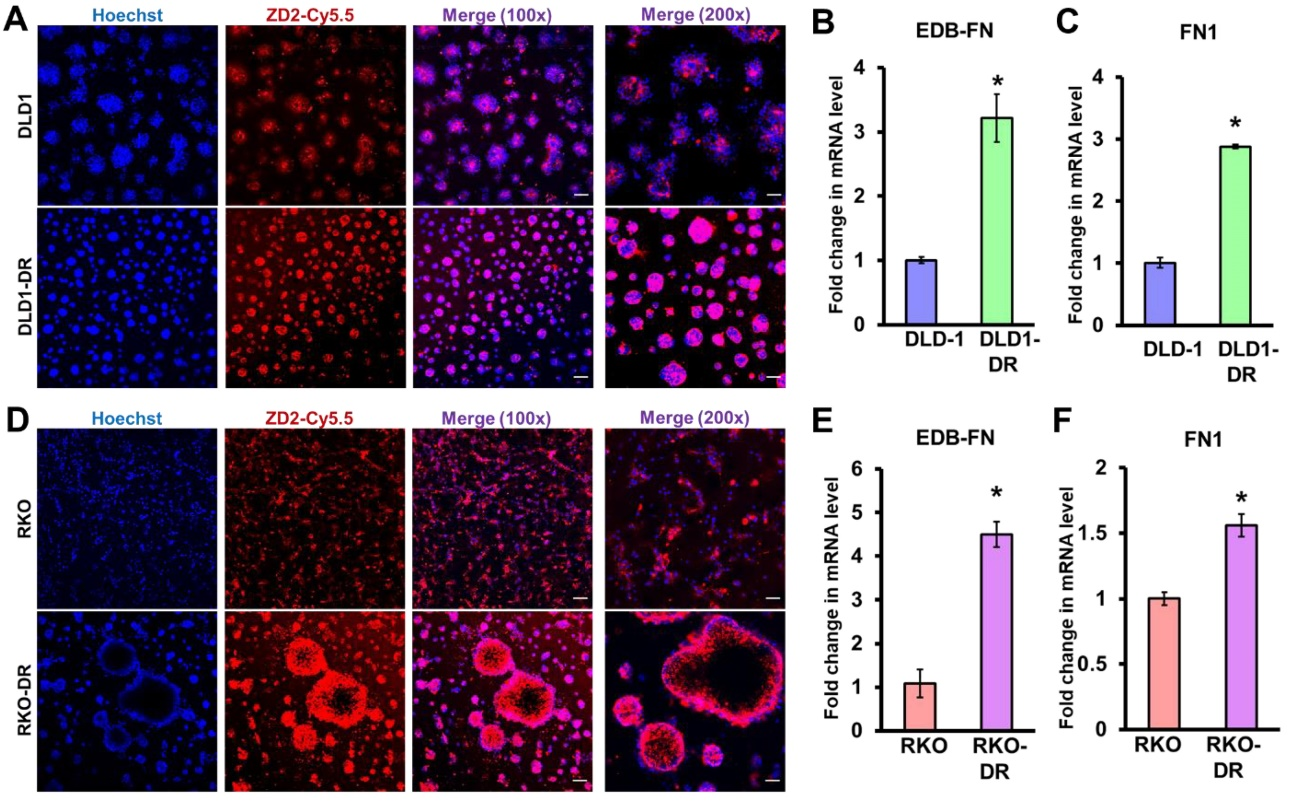
** Acquired drug resistance is associated with EDB-FN expression in CRC cells.** CRC cells cultured in 3D Matrigel were stained with 100 nM EDB-FN-specific peptide probe ZD2-Cy5.5 and 5 µg/mL nuclear stain Hoechst. CRC cells grown in 2D monolayer culture were harvested for RNA extraction and qRT-PCR analysis of mRNA expression. Drug-resistant DLD1-DR cells show** A.** increased ZD2-Cy5.5 binding, and 3-fold higher mRNA levels of **B.** EDB-FN and **C.** FN1, compared to DLD-1 cells. Drug-resistant RKO-DR cells show** D.** increased ZD2-Cy5.5 binding, and 4- and 1.5-fold higher mRNA levels of** E.** EDB-FN and **F.** FN1, compared to RKO cells, respectively. Bars indicate mean ± sem. *p<0.05 using unpaired *t*-test. Scale bar = 100 µm (100x) and 50 µm (200x).

As shown in **Figure 2A**, both DLD-1 and DLD1-DR cell lines expressed EDB-FN, evident in their ZD2-Cy5.5 binding. However, the DLD1-DR cells showed brighter fluorescence signal, indicating higher ZD2-Cy5.5 binding, which was confirmed by the 3-fold upregulation of EDB-FN mRNA level over DLD-1 cells** (Figure 2B)**. Total FN1 expression was also found to increase with drug resistance in DLD1-DR cells (ca. 3-fold,** Figure 2C**). Similarly, while both RKO and RKO-DR cells secreted EDB-FN, its expression was significantly higher in the latter, reflected in the intense ZD2-Cy5.5 staining **(Figure 2D)** and approximately 4.5-fold increase in EDB-FN mRNA **(Figure 2E)**, over the former. Total FN1 was only increased by 1.5-fold in the RKO-DR cells** (Figure 2F)**. These results demonstrate that acquired drug resistance in CRC cells is associated with EDB-FN overexpression.

### Contrast-enhanced MRMI of EDB-FN using MT218 facilitates effective differential diagnosis of drug-resistant CRC tumors

To determine whether EDB-FN overexpression can be used as a molecular marker to differentiate between non-resistant and drug-resistant CRC tumors, MRMI was performed using EDB-FN-targeting contrast agent MT218 51 in athymic nu/nu mice bearing subcutaneous xenografts of DLD-1, DLD1-DR, RKO, and RKO-DR. T_1_-weighted coronal and axial images were acquired before and 25 min after injection of 40 µmol/kg MT218. We previously showed that this subclinical dose of MT218 is just as effective as the standard dose (0.1 mmol/kg) of MT218 and the clinical contrast agent Gadoteridol 53.

**Figure 3:**
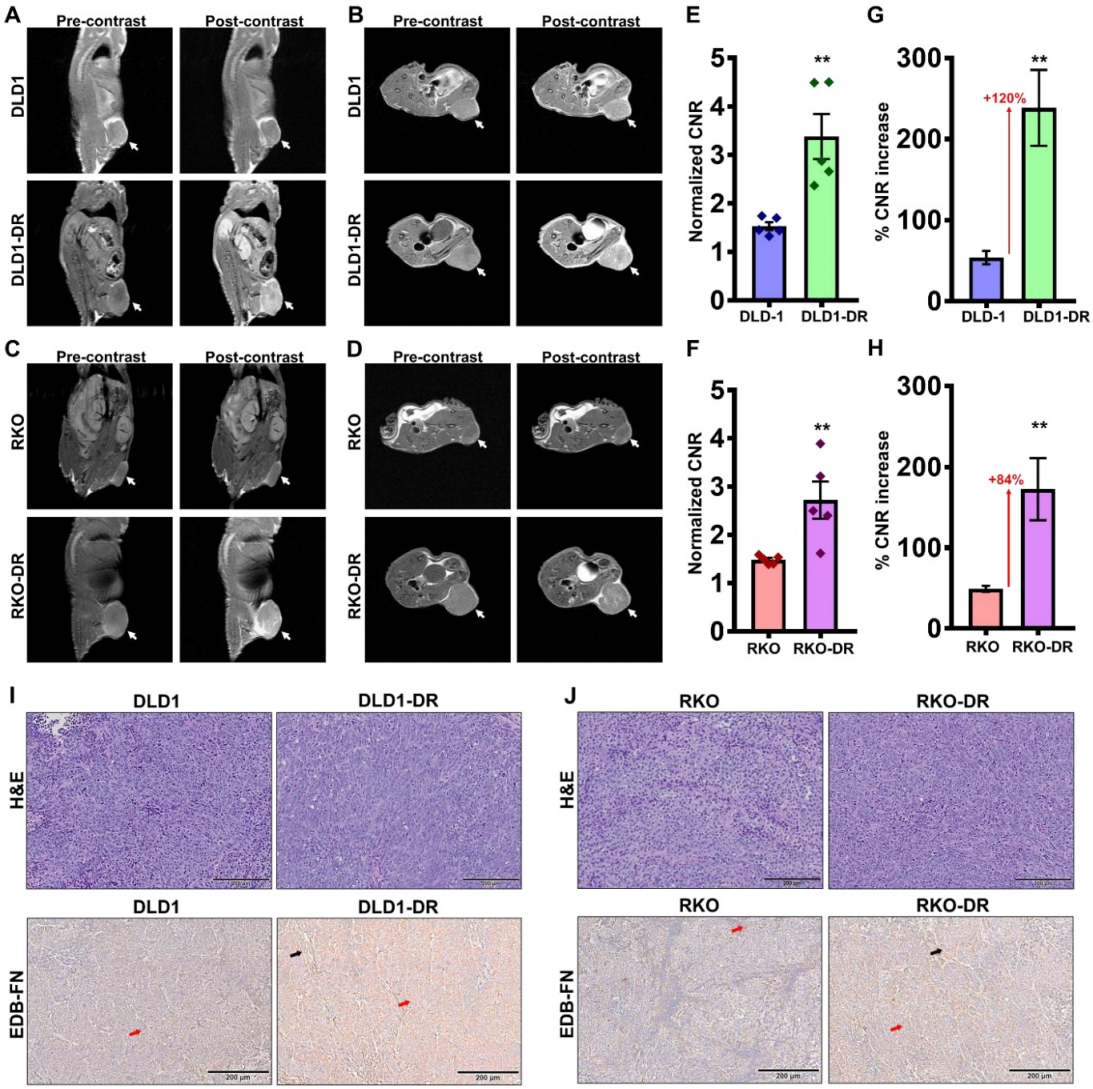
** Contrast-enhanced MRMI of EDB-FN using MT218 facilitates non-invasive assessment of drug resistance in CRC tumors.** T_1_-weighted FSE coronal and axial images were obtained pre- and post-injection (25 min) of 40 µmol/kg dose of MT218 in CRC xenograft-bearing mice. Representative coronal and axial images of MRI show robust signal enhancement in **A-B.** DLD1-DR and **C-D.** RKO-DR tumors, compared to the non-resistant DLD-1 and RKO tumors (white arrows: tumors). Drug-resistant **E-F.** DLD1-DR and **G-H.** RKO-DR tumors exhibit significantly higher CNRs over DLD-1 and RKO tumors, respectively (n=5 mice per group). Bars indicate mean ± sem and dots indicate individual mouse CNRs. *p<0.05 using unpaired *t*-test. Post-mortem histology shows poorly differentiated mitotically active cells by H&E staining, and stronger anti-EDB-FN antibody G4 staining in drug-resistant **I.** DLD1-DR and **J.** RKO-DR tumor sections over their respective non-resistant counterparts (red and black arrows denote EDB-FN in tumor cells and tumor-associated fibroblasts, respectively).

Preliminary time course analysis showed robust enhancement and increased CNRs up to 35 min post-injection of MT218 in both the DLD-1** (Figure S2A-D)** and RKO **(Figure S3A-D)** tumor models. Since the peak enhancement was observed between 20-35 min, the 25 min time point was selected for the subsequent assessment of drug resistance. MRMI of EDB-FN using MT218 resulted in signal enhancement in the non-resistant DLD-1 and RKO tumors **(Figure 3A-D)**, with 1.53- and 1.4-fold increase in CNRs over the pre-contrast, **(Figure 3E-F)**, respectively. The drug-resistant DLD1-DR and RKO-DR tumors exhibited significantly stronger signal enhancement, reflected in the 3.3- and 2.6-fold higher CNRs over pre-contrast **(Figure 3A-F)**. Importantly, this signal enhancement was more robust than in their non-resistant counterparts, resulting in 2.2- and 1.8-fold higher CNRs (i.e., 120% and 84% increase) over the non-resistant DLD-1 and RKO tumors, respectively **(Figure 3E-H)**. The mice were euthanized post-imaging and their tumor tissues were processed for H&E staining and EDB-FN IHC with G4 antibody. As shown in **Figure 3I-J**, H&E staining did not show significant differences between the drug-resistant and non-resistant CRC tumors, with both demonstrating high grade, poorly differentiated, and mitotically active pleomorphic cancer cells. IHC for EDB-FN exhibited stronger staining in DLD1-DR **(Figure 3I)** and RKO-DR** (Figure 3J)** tumors, compared to that in the DLD-1 and RKO tumors, respectively. EDB-FN staining was localized in both the tumor cells and the elongated spindle-like interspersed cancer-associated fibroblasts (CAFs), denoted by the red and black arrows, respectively. This IHC data correlated with the signal enhancement and CNRs observed in the four models, demonstrating that MRMI of EDB-FN with a reduced dose of MT218 can facilitate non-invasive assessment of drug resistance in CRC models.

### MRMI of EDB-FN using MT218 facilitates therapeutic response monitoring of drug-resistant CRC tumors

**Figure 4:**
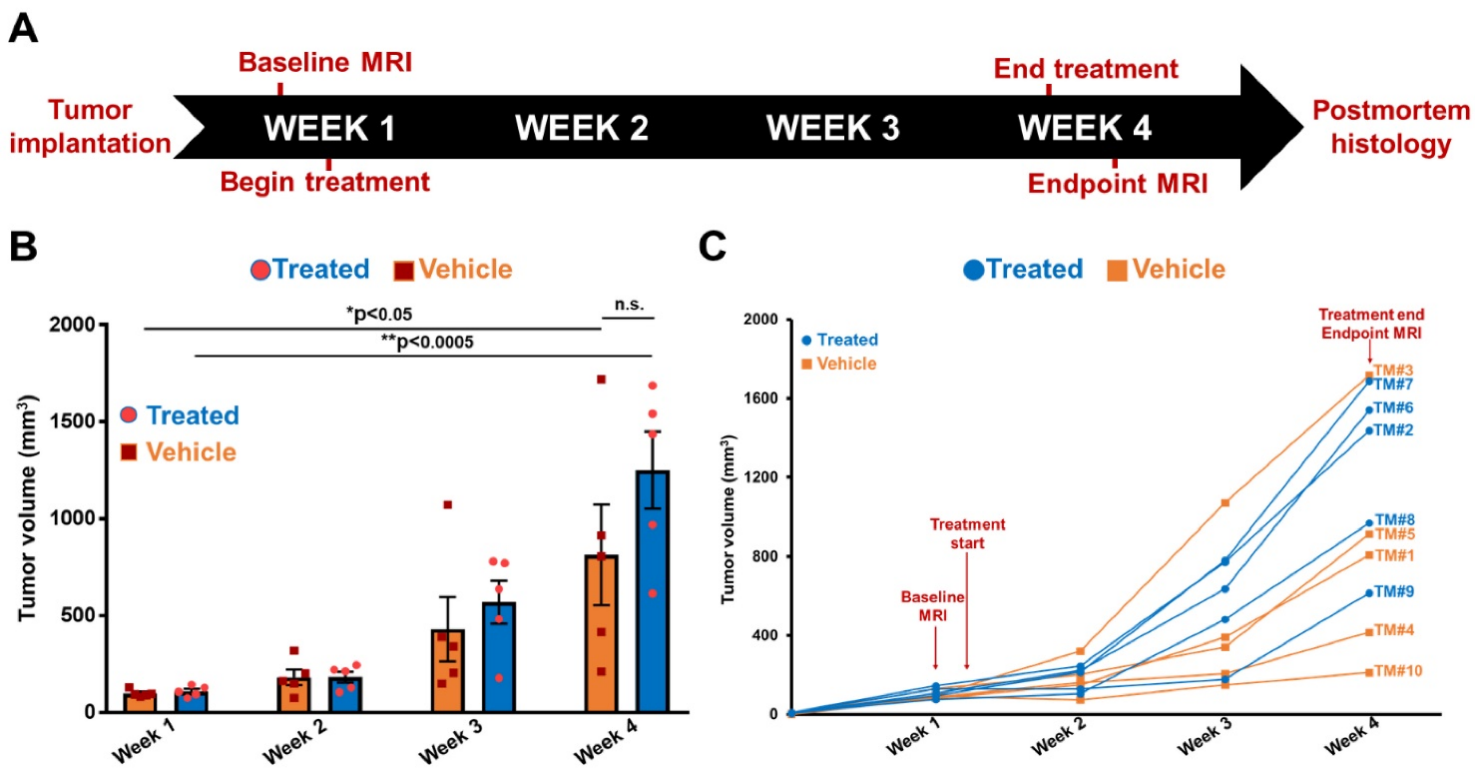
** Timeline of therapeutic regimen and non-invasive monitoring of therapeutic efficacy in drug-resistant DLD1-DR tumor-bearing mice. A.** Drug-resistant DLD1-DR tumors were subcutaneously implanted in the flanks of 10 athymic nu/nu female mice. Tumor volumes were monitored weekly with Vernier caliper. Baseline MRMI for EDB-FN using MT218 was performed at week 1, when the tumors reached 100 mm^3^ volumes. Mice were randomized into DMSO-treated 'vehicle' and MK2206-treated 'treated' groups (n=5 each). At week 4, endpoint MRMI for EDB-FN using MT218 was performed and the experiment was terminated for post-mortem histology.** B.** Average tumor volumes increase from week 1 to 4 for each group.** C.** Tumor volumes for individual mice (numbered TM1-TM10) from both the vehicle and treated groups increase progressively from week 1 to 4. (n=5 mice per group). Bars indicate mean ± sem and dots indicate individual mouse tumor volumes. *p<0.05 using unpaired *t*-test.

Following differential diagnosis of drug-resistant CRC tumors, the potential of MRMI of EDB-FN for non-invasive assessment of therapeutic response was determined in DLD1-DR-bearing mice treated with MK2206-HCl, a pan-AKT inhibitor. **Figure 4A** shows the schematic of the timeline of therapeutic regimen and MRMI for therapeutic efficacy monitoring. One week after tumor implantation, baseline MRMI was performed, followed by randomization of the mice into vehicle (DMSO-treated: TM1, TM3, TM4, TM5, TM10) and treated (MK2206-treated: TM2, TM6, TM7, TM8, TM9) groups. Mice were treated once a week for 3 weeks, and endpoint MRMI was performed at week 4 due to high tumor burden, followed by post-mortem histology. Tumor volumes were monitored once a week. **Figure 4B** shows the progression of tumor growth over the 4-week period. The average tumor volumes of both vehicle and treated groups increased significantly, from 97.7 ± 8.14 mm^3^ to 813.4 ± 259.7 mm^3^ and from 110.5 ± 11.97 mm^3^ to 1249.7 ± 199.3 mm^3^, respectively. Although no significant difference was found between the average tumor volumes of the vehicle and treated groups (p=0.22) at week 4, individual mice in the latter group showed visibly worse and larger tumors, indicating that the drug-resistant tumors did not respond to therapy. The tumor volumes of individual mice were also monitored **(Figure 4C)** to correlate with the endpoint MRMI signal and EDB-FN expression. Each mouse (labeled from TM1-TM10) showed significant increase in tumor volumes, to a lesser or greater extent.

**Figure 5:**
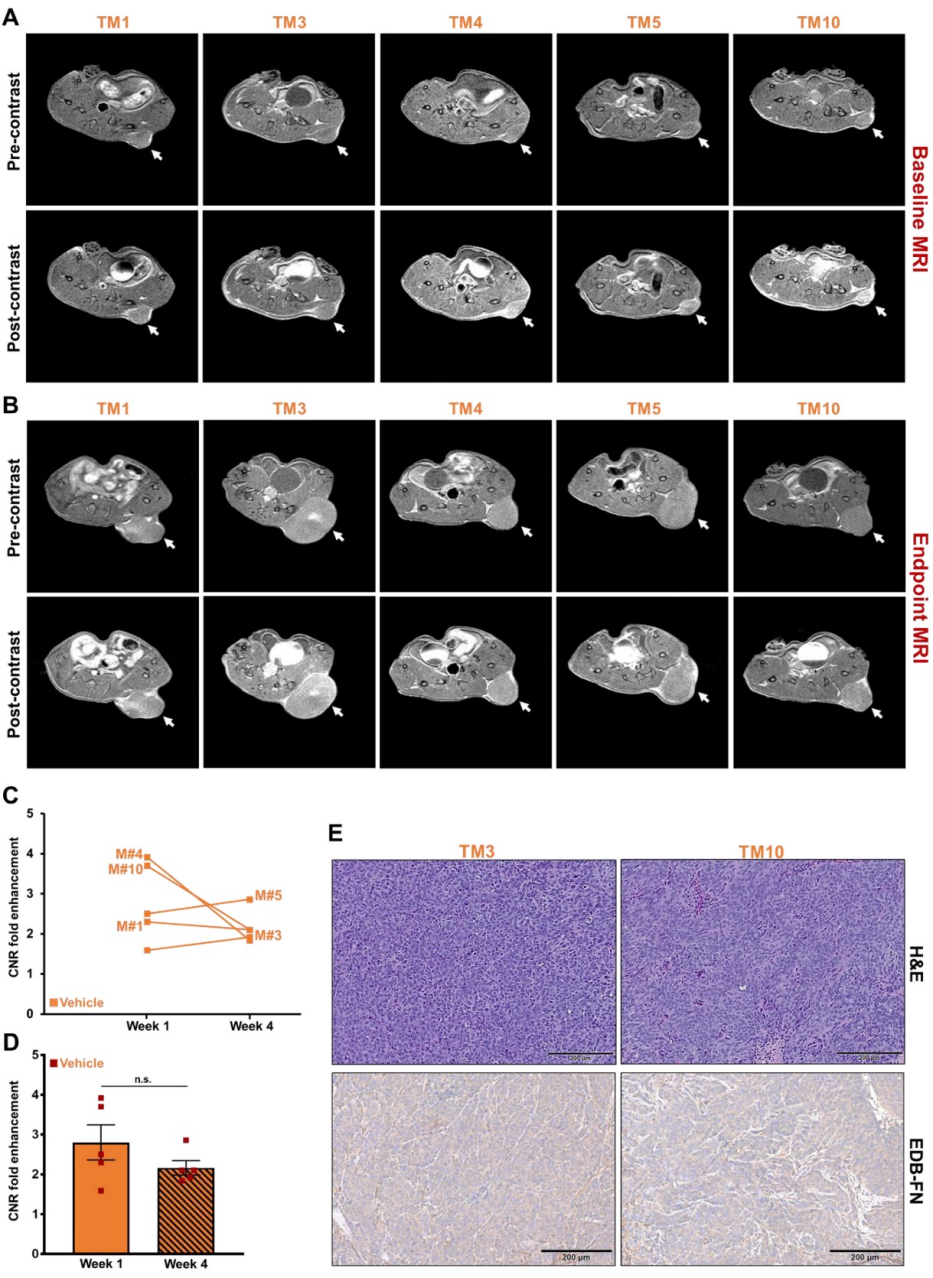
** MRMI of EDB-FN with MT218 facilitates non-invasive therapeutic monitoring of vehicle-treated mice bearing drug-resistant CRC tumors.** T_1_-weighted 2D spin echo axial images from MRMI performed at** A.** Baseline (week 1) and **B.** Endpoint (week 4) with 40 µmol/kg dose MT218 in DLD1-DR-bearing mice treated with DMSO (TM: designation for individual mice in vehicle group). Images were taken before and 25 min post-injection of MT218. **C.** CNRs for individual mice plotted for week 1 and week 4.** D.** Average CNRs for the vehicle group did not change significantly from week 1 to week 4. **E.** Postmortem histology performed after week 4 shows poorly differentiated DLD1-DR tumor sections. Immunohistochemistry with anti-EDB-FN antibody G4 shows stronger staining in TM3 than TM10. (n=5 mice per group). Bars indicate mean ± sem and dots indicate individual mouse tumor volumes. *p<0.05 using unpaired *t*-test.

The tumor growth was monitored with endpoint MRMI for EDB-FN using MT218. As shown in **Figure 5A**, each mouse in the vehicle group showed signal enhancement compared to pre-contrast, when baseline MRMI was performed. After 3 weeks of DMSO injections, all the 5 mice showed signal enhancement in endpoint MRMI; with mice # TM3 and TM5 showing increased signal enhancement and tumor size, compared to the other 3 mice (TM1, TM4, and TM10), **Figure 5B**. Quantification of this data showed a consistent trend, with mice # TM3 and TM5 showing increase in CNRs from week 1 to 4, and mice # TM1, TM4, and TM10 showing decrease in CNRs from week 1 to 4 **(Figure 5C)**. The MRMI signal also correlated with the increase in their tumor volumes, where mice # TM3 and TM5 showed a rapid increase in tumor volumes compared to mice # TM1, TM4, and TM10 **(Figure 4C)**. No significant difference was found between the average CNRs at week 1 and 4 for the vehicle group** (Figure 5D)**. Postmortem histology and IHC for EDB-FN showed stronger G4 staining in TM3 than TM10, demonstrating MRMI of EDB-FN with MT218 correlates with the endogenous tumor EDB-FN expression **(Figure 5E)**.

**Figure 6:**
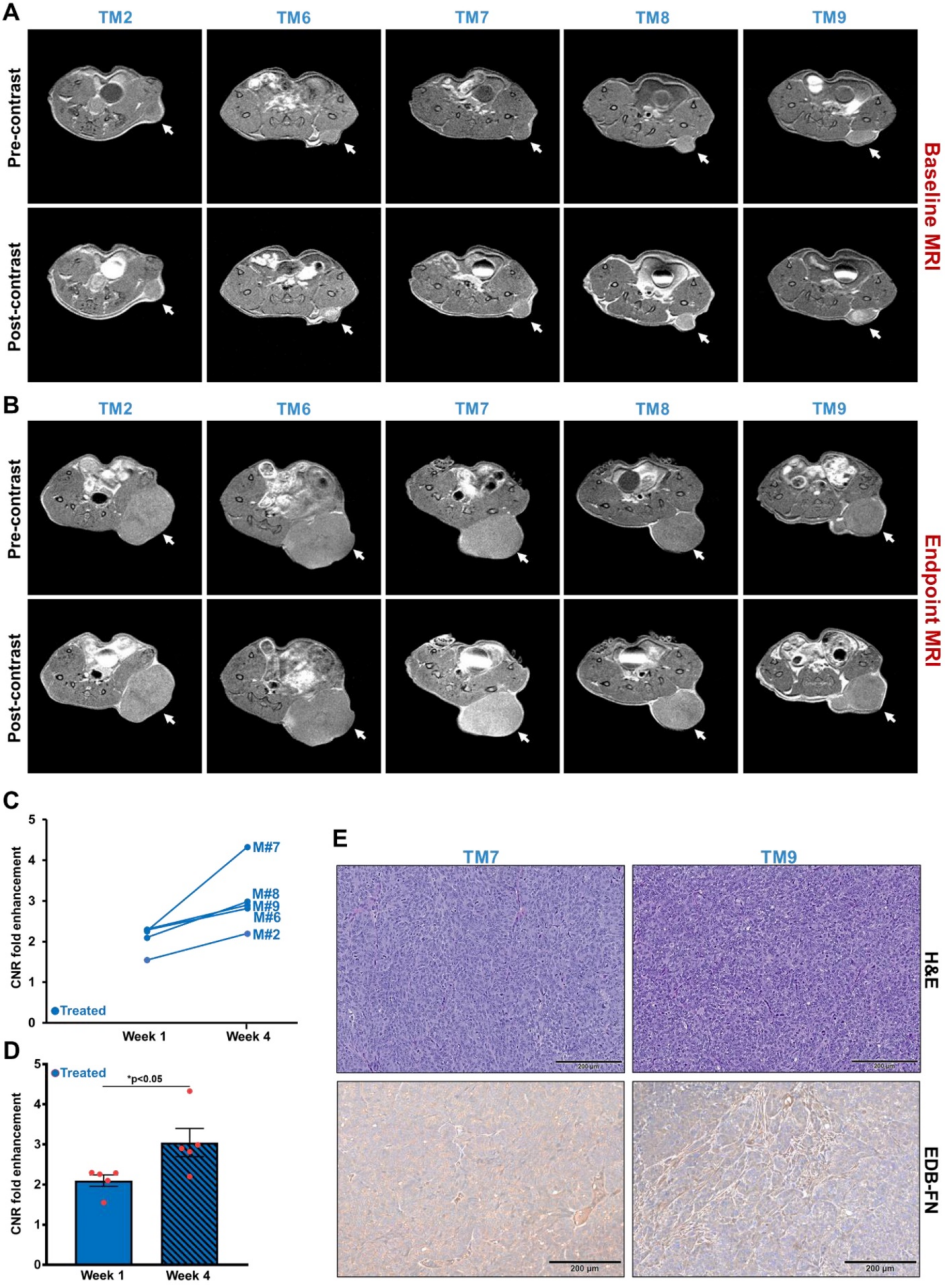
** MRMI of EDB-FN with MT218 facilitates non-invasive therapeutic monitoring of MK2206-HCl-treated mice bearing drug-resistant CRC tumors.** T_1_-weighted 2D spin echo axial images from MRMI performed at **A.** Baseline (week 1) and **B.** Endpoint (week 4) with 40 µmol/kg dose MT218 in DLD1-DR-bearing mice treated with 100 mg/kg MK2206-HCl (TM: designation for individual mice in treated group). Images were taken before and 25 min post-injection of MT218.** C.** CNRs for individual mice plotted for week 1 and week 4. **D.** Average CNRs for the treated group increase significantly from week 1 to week 4.** E.** Postmortem histology performed after week 4 shows poorly differentiated DLD1-DR tumor sections. Immunohistochemistry with anti-EDB-FN antibody G4 shows stronger staining in TM7 than TM9. (n=5 mice per group). Bars indicate mean ± sem and dots indicate individual mouse tumor volumes. *p<0.05 using unpaired *t*-test.

As for the treated group, each of the 5 mice showed high signal enhancement over pre-contrast during baseline MRMI **(Figure 6A)**. Despite 3 weeks of treatment, the drug-resistant DLD1-DR tumors failed to respond to therapy and increased in size with increased signal enhancement over pre-contrast **(Figure 6B)**, reflected in the elevated CNRs for the individual mice from week 1 to week 4** (Figure 6C)**. Among the 5 mice, mouse # TM7 showed the highest signal enhancement and CNR, and the largest tumor volume** (Figure 4C)**. On the other hand, mice # TM2, TM6, TM8, and TM9 showed increased CNRs and tumor volumes from week 1 to 4, but no correlating pattern between the two was detected. The average tumor CNRs in the treated group increased significantly from week 1 to 4** (Figure 6D)**, confirming that the drug-resistant tumors failed to respond to therapy, and performed worse than the vehicle group. Postmortem histology and IHC for EDB-FN showed strong G4 staining for the tumor in mouse # TM7, compared to TM9 **(Figure 6E)** as well as vehicle-treated mice # TM3 and TM10 **(Figure 5E)**, further corroborating the direct correlation of EDB-FN expression and MRMI-based monitoring. While the week 4 average CNR of the treated group was higher than that of the vehicle group, it was not statistically significant (p=0.055, **Figure S4**). Taken together, these results demonstrate that MRMI of EDB-FN with MT218 can facilitate non-invasive therapeutic monitoring of drug-resistant CRC tumors.

### EDB-FN is overexpressed in human colon adenocarcinoma and is correlated with poor patient survival

**Figure 7:**
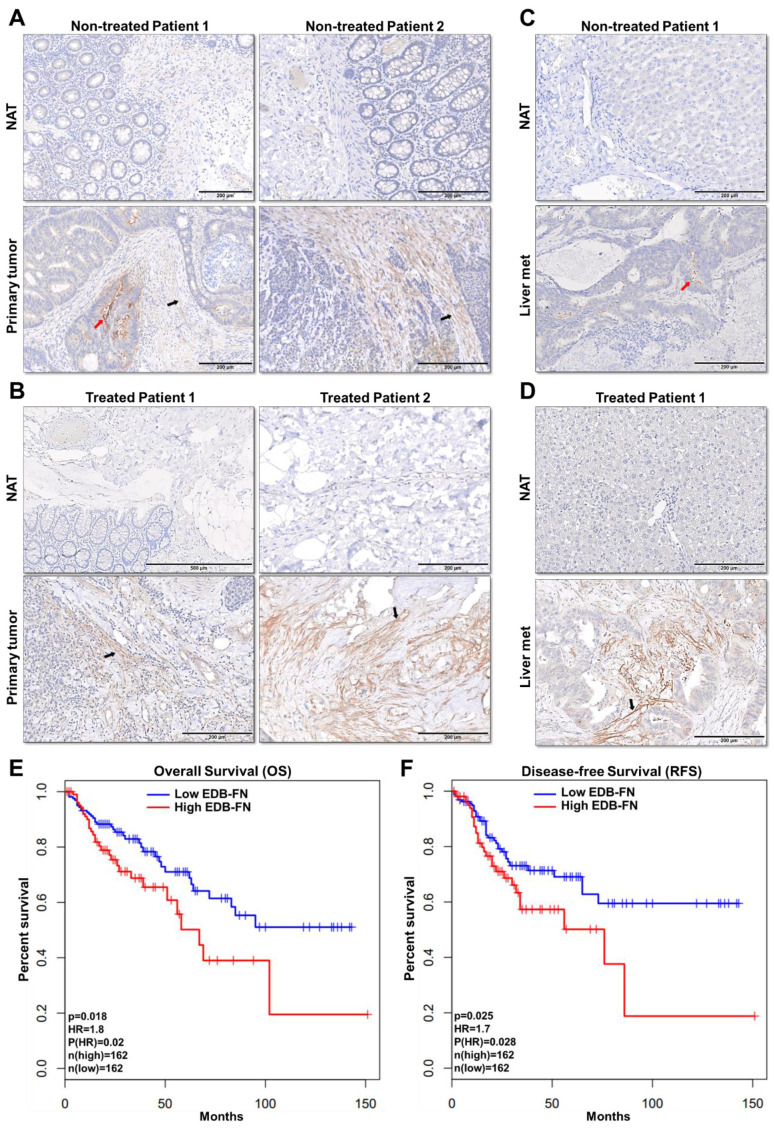
** EDB-FN is overexpressed in human colon adenocarcinoma and is correlated with poor survival.** Immunohistochemical staining using EDB-FN-specific antibody G4 shows strong EDB-FN expression in representative primary colon adenocarcinoma tissues from **A.** non-treated patients and **B.** treated patients, but negligible EDB-FN expression in the corresponding normal adjacent tissues (NAT). Immunohistochemical staining of representative metastatic liver specimens shows differential EDB-FN expression in **C.** non-treated and **D.** treated patients, with no expression in the corresponding normal adjacent liver tissue (NAT). Black and red arrows denote fibroblasts and tumor cells, respectively. Kaplan-Meier curves demonstrate that high EDB-FN levels correlate with **E.** poor overall survival (OS) and **F.** poor disease-free survival (RFS) of colon adenocarcinoma patients (*p=0.018 and *p=0.02 for Hazard ratio for OS and *p=0.025 and *p=0.028 for RFS using Log-rank test, i.e., Mantel-Cox test).

To assess the feasibility of using MRMI of EDB-FN in CRC patients, the expression of EDB-FN was analyzed in representative human specimens of colon adenocarcinoma (COAD), metastatic livers, and their corresponding normal adjacent tissues. Primary COAD tumors were found to demonstrate strong EDB-FN expression in both non-treated **(Figure 7A)** and treated** (Figure 7B)** patients, with negligible expression in the corresponding normal adjacent tissues (NAT). On the other hand, COAD hepatic metastases showed differential EDB-FN levels, with moderate staining in non-treated patient **(Figure 7C)** and intense staining in treated patient **(Figure 7D)**, and no staining in normal adjacent liver. In regards to the pattern, most of the EDB-FN staining was observed in the stromal and cancer-associated fibroblasts (black arrows), and some in the tumor cells (red arrows), in the primary and metastatic sites. Moreover, analysis of RNA-Seq data from TCGA and GTEx databases revealed that increased EDB-FN levels correlated with poor overall survival (OS) **(Figure 7E)** and poor disease-free survival (RFS) **(Figure 7F)** of COAD patients, suggesting that EDB-FN overexpression could be a prognostic indicator for COAD.

## Discussion

This work demonstrates that invasive drug-resistant CRC tumors overexpress the ECM oncoprotein EDB-FN, compared to their non-resistant counterparts. Drug-resistant CRC tumors that show negative response to targeted therapy also upregulate EDB-FN. These therapeutic pressure-induced alterations in EDB-FN levels enable effective non-invasive assessment and treatment response monitoring of drug-resistant CRC by MRMI, even at subclinical doses of MT218.

The molecularly diverse landscape of CRC underscores the need for robust oncomarkers that can provide diagnostic, prognostic, or therapy-predictive value 59. Here, we showed, for the first time, that overexpression of EDB-FN correlated negatively with CRC patient survival. A prior study that showed poor prognosis of post-operative CRC patients with high oncFN1 levels used FDC-6 antibody, which binds to an alternative oncofetal isoform of fibronectin 60. Immunohistochemical analysis of patient samples showed strong EDB-FN-specific staining with G4 antibody in the primary and metastatic CRC sites. EDB-FN was found to be localized in the stroma, stromal fibroblasts, adenocarcinoma cells, and fibroblasts interspersed around these tumor cells. These results validate the recently emerging consensus that multiple cell types in the tumor milieu express EDB-FN 42, 43, suggesting multifaceted interactions of the cellular and extracellular components of the tumor microenvironment. The profound tumor-specific expression of EDB-FN was evident from the complete absence of G4 staining in the normal adjacent colon and liver tissues, signifying EDB-FN as an attractive candidate marker for CRC. The detection of over 300% increase in EDB-FN levels in the urinary samples of muscle-invasive bladder cancer patients, and a negative correlation to their clinical outcomes 41, also validates its potential as a promising diagnostic oncomarker for other cancers.

Both the 2D and 3D cell cultures demonstrated a positive association between the invasiveness of drug-resistant CRC cells and their endogenous EDB-FN expression. Between the two non-drug-resistant models, the DLD-1 cells exhibited higher expression of EDB-FN than the RKO cells. This is consistent with the inherent biological properties of the models, wherein the DLD-1 cells derived from Duke's type C, p53-mutated, CEA-expressing adenocarcinoma are more invasive and migratory than the RKO cells derived from a p53-, K-Ras-wild type primary carcinoma site 61, 62. Development of resistance to 5ʹ-FU by DLD-1 and 5ʹ-FU + CB-839 by RKO cells resulted in dynamically altered EMT-like DLD1-DR and hybrid E-M RKO-DR cells, highlighting the distinct trajectories taken by the two cell lines following therapeutic pressure-induced clonal selection 22. Both the drug-refractory cell lines also exhibited robust overexpression of the ATP-binding cassette (ABC) transporter MDR-1, which is unsurprising given that enhanced cellular efflux is a common mechanism of 5ʹ-FU-resistance 58. It is likely that the increased plasticity induced by the hybrid E-M phenotype confers CB-839 resistance on the RKO-DR cells 58. Enhanced plasticity and metabolic redundancy have been previously observed in CB-839-resistant breast cancer 63, 64, although the precise mechanisms of glutaminase inhibition resistance in CRC remain understudied.

Irrespective of the heterogeneous molecular changes and EMT status, acquisition of invasive and migratory properties by both the drug-resistant CRC cell lines was associated with significant upregulation of EDB-FN. This upregulation was recapitulated in the drug-resistant DLD1-DR and RKO-DR xenografts, which exhibited robust signal enhancement and significantly higher CNRs in MRMI with EDB-FN-targeting contrast agent MT218, over their respective non-resistant counterparts, suggesting the potential of EDB-FN as a promising diagnostic oncomarker. We previously showed overexpression of EDB-FN in drug-resistant breast cancer cells 45. Three other groups also demonstrated elevated EDB-FN in CRC cells and tumors 42, 43, 65. To our knowledge, this is the first report demonstrating EDB-FN overexpression in drug-refractory CRC. These elevated EDB-FN levels could be leveraged for ZD2-targeted MRMI-mediated non-invasive surveillance of tumor progression, to identify critical events and stages that precede the development of drug resistance.

Drug resistance is the bane of oncotherapy and cancer radiology. A common strategy for treating disease relapse in patients involves adaptive interventions with a different class of drugs to counteract the new driver mutation profiles, which can vary between patients 66. Accurate non-invasive assessment of these drug-resistant cells and tumor response to oncotherapy (early within few days of treatment) has the potential to revolutionize the contemporary cancer treatment paradigm. Multiple lines of clinical evidence now indicate that assessment of therapeutic response cannot be based on the change of tumor size alone 26, 67. Consequently, we hypothesized that molecular imaging of abundantly overexpressed EDB-FN in correlation with the aggressive nature of the disease and independent of tumor size can potentially provide accurate tumor response to therapies. In our treatment regimen, the failure of the highly drug-resistant DLD1-DR tumors to MK2206-HCl therapy was reflected in their increased EDB-FN levels, and a consequent increase in MT218 uptake, demonstrated by robust signal enhancement and increased CNRs in MRMI. These tumors also increased in size, as did the vehicle tumors; however, the latter did not show comparable increase in EDB-FN and MT218 uptake as the treated tumors, suggesting that the MRMI was based on inherent EDB-FN expression and not anatomical tumor size. While the negative response to MK2206-HCl was unexpected considering our previous findings on the role of phospho-AKT signaling in EDB-FN regulation 45 in breast cancer cells, we speculate that the enhanced plasticity and overexpression of the drug efflux pump MDR-1 could have rendered the DLD1-DR tumors non-responsive to MK2206-HCl. A compensatory activation of alternative AKT-independent oncogenic signaling (e.g., NF-κβ) in 5ʹ-FU-treated DLD-1 cells has also been shown before 68. The treatment was terminated at week 4 due to the large tumor burden, precluding any other adaptive interventions or imaging tests. Although this research was limited to the imaging of primary tumors, the promising results warrant further investigation in to analyzing additional parameters and imaging modalities in conjunction with monitoring tumor volumes to validate and correlate the tumor responses to various therapies.

Only two previous reports on EDB-FN-based radioimmunotherapy in CRC using ^125^I-labeled L19-SIP antibody exist, which demonstrated selective tumor uptake and tumor growth inhibition in CRC xenografts 65 and CRC patients 42; however, EDB-FN-based MRMI assessment of drug-resistant CRC has never been performed before. Our preliminary data serve as a groundwork for transforming EDB-FN-targeted molecular imaging, alone and as an adjunct to other screening modalities, into a clinically viable technology for CRC management. One of the challenges of MRI in CRC imaging is accurate detection of hepatic CRC metastases, a major prognostic indicator of patient outcomes 8. Given that we and independent researchers 42 observed elevated EDB-FN expression in patient metastatic liver specimens, accurate and timely detection of these evasive niches could provide a crucial diagnostic advantage during treatment regimens. Although MRI is the primary modality of choice for staging rectal cancer, it's performance for tumor restaging following oncotherapy is inconsistent 6. Therefore, it would be interesting to evaluate the efficacy of MRMI with MT218 in determining the response of patients to neoadjuvant therapy during the “wait and watch” period, before planning surgical interventions.

Although contrast-enhanced MRI remains crucial for diagnosis, cumulative exposure to contrast media, especially linear GBCAs, results in deposition and long-term accumulation of Gd in the brain, bones, and even skin of patients 20, 69, 70. MT218 is a small peptide conjugate of a clinical macrocyclic contrast agent Gadoteridol with high stability and good safety profile. We recently demonstrated strong tumor-specific enhancement in MRMI with MT218 at a reduced dose of 40 µmol/kg in comparison to the standard clinical dose of 0.1 mmol/kg in breast cancer models 53. The uncompromised efficacy of MRMI with MT218 at the reduced dose can minimize the potential risk of Gd-associated and dose-dependent toxicity. This low dose was also sufficient to enable effective assessment of CRC and drug-resistant CRC tumors in this study. By virtue of the high T_1_ relaxivity (6.13 mM^-1^s^-1^ at 3T) of MT218, effective MRMI at subclinical doses, and easy accessibility of abundant EDB-FN in the tumor microenvironment to MT218 binding, we posit that MRMI with reduced Gd exposure is promising for active surveillance and treatment monitoring of CRC. Given that tumor cell plasticity engenders extensive patient-to-patient variability in tumor progression and therapeutic response, even within the same cancer type 22, MRMI with MT218 based on the tumor levels of EDB-FN could detect the development of drug resistance early during the chemotherapeutic period and also to adaptive therapies, helping to tailor treatment regimen for the relevant patients and improving the success of oncotherapy.

The unique advantages of EDB-FN-specific ZD2 peptide can also be harnessed to develop integrated imaging systems like PET/MRI or PET/CT for multi-parametric molecular imaging. Indeed, PET and SPECT probes have already been generated by conjugating the EDB-FN-specific ZD2 peptide to radiotracers like ^64^Cu-DOTA, ^68^Ga-NOTA, and ^99m^Tc-HYNIC chelates for improved detection of prostate, pancreatic, and breast cancers 48, 71, 72, respectively. These systems could also provide insight into several aspects of CRC, including metastatic surveillance following primary tumor resection, imaging of CRC in syngeneic models treated with chemotherapy, and assessment of tumor response to immunotherapy. Besides ZD2, other EDB-FN-targeting ligands, antibodies and nanobodies, including APT-_FN-EDB_, L19, BC1, and NJB2 have also been used to deliver therapeutic and imaging agents to tumor ECM and vasculature 46, 73-75, validating the role of EDB-FN as a promising oncomarker.

In summary, we demonstrate tumor-specific EDB-FN expression in COAD specimens, and poor prognosis of COAD patients with high levels of EDB-FN. While CRC cells and tumor xenografts inherently express EDB-FN, its expression is further elevated in their drug-resistant counterparts. MRMI of EDB-FN with a significantly reduced dose of MT218 can facilitate effective non-invasive assessment and therapeutic monitoring of drug-resistant CRC, highlighting the translational potential of MT218-mediated EDB-FN-targeting MRMI in active surveillance and monitoring of drug-resistant neoplasms.

## Supplementary Material

Supplementary figures.

## Abbreviations

COAD: colon adenocarcinoma
CNR: contrast-to-noise ratio
CRC: colorectal cancer
ECM: extracellular matrix
EDB-FN: extradomain-B fibronectin
EMT: epithelial-mesenchymal transition
GBCA: gadolinium-based contrast agent
5ʹ-FU: 5ʹ-fluorouracil
GTEx: genotype-tissue expression
H&E: hematoxylin & eosin
IHC: immunohistochemistry
MDR1: multidrug resistance 1
MRMI: magnetic resonance molecular imaging
PET-CT: positron emission tomography-computed tomography
SPECT: single-photon emission computed tomography
TCGA: the cancer genome atlas

## References

[1] Siegel RL, Miller KD, Jemal A (2019). Cancer statistics, 2019. CA Cancer J Clin.

[2] Cronin KA, Lake AJ, Scott S, Sherman RL, Noone AM, Howlader N (2018). Annual Report to the Nation on the Status of Cancer, part I: National cancer statistics. Cancer.

[3] Knudsen AB, Zauber AG, Rutter CM, Naber SK, Doria-Rose VP, Pabiniak C (2016). Estimation of Benefits, Burden, and Harms of Colorectal Cancer Screening Strategies: Modeling Study for the US Preventive Services Task Force. JAMA.

[4] Shaukat A, Mongin SJ, Geisser MS, Lederle FA, Bond JH, Mandel JS (2013). Long-term mortality after screening for colorectal cancer. N Engl J Med.

[5] Tirumani SH, Kim KW, Nishino M, Howard SA, Krajewski KM, Jagannathan JP (2014). Update on the role of imaging in management of metastatic colorectal cancer. Radiographics.

[6] McKeown E, Nelson DW, Johnson EK, Maykel JA, Stojadinovic A, Nissan A (2014). Current approaches and challenges for monitoring treatment response in colon and rectal cancer. J Cancer.

[7] Eisenhauer EA, Therasse P, Bogaerts J, Schwartz LH, Sargent D, Ford R (2009). New response evaluation criteria in solid tumours: revised RECIST guideline (version 1.1). Eur J Cancer.

[8] Van Cutsem E, Verheul HM, Flamen P, Rougier P, Beets-Tan R, Glynne-Jones R (2016). Imaging in Colorectal Cancer: Progress and Challenges for the Clinicians. Cancers (Basel).

[9] Marcus CD, Ladam-Marcus V, Cucu C, Bouche O, Lucas L, Hoeffel C (2009). Imaging techniques to evaluate the response to treatment in oncology: current standards and perspectives. Crit Rev Oncol Hematol.

[10] Pellino G, Gallo G, Pallante P, Capasso R, De Stefano A, Maretto I (2018). Noninvasive Biomarkers of Colorectal Cancer: Role in Diagnosis and Personalised Treatment Perspectives. Gastroenterol Res Pract.

[11] Tie J, Cohen JD, Wang Y, Christie M, Simons K, Lee M (2019). Circulating Tumor DNA Analyses as Markers of Recurrence Risk and Benefit of Adjuvant Therapy for Stage III Colon Cancer. JAMA Oncol.

[12] Osumi H, Shinozaki E, Yamaguchi K, Zembutsu H (2019). Early change in circulating tumor DNA as a potential predictor of response to chemotherapy in patients with metastatic colorectal cancer. Sci Rep.

[13] Griffeth LK (2005). Use of PET/CT scanning in cancer patients: technical and practical considerations. Proc (Bayl Univ Med Cent).

[14] Maeda C, Endo S, Mori Y, Mukai S, Hidaka E, Ishida F (2019). The ability of positron emission tomography/computed tomography to detect synchronous colonic cancers in patients with obstructive colorectal cancer. Mol Clin Oncol.

[15] Jhaveri KS, Hosseini-Nik H (2015). MRI of Rectal Cancer: An Overview and Update on Recent Advances. AJR Am J Roentgenol.

[16] Garcia-Figueiras R, Baleato-Gonzalez S, Padhani AR, Marhuenda A, Luna A, Alcala L (2016). Advanced imaging of colorectal cancer: From anatomy to molecular imaging. Insights Imaging.

[17] Xu K, Xu C, Zhang Y, Qi F, Yu B, Li P (2018). Identification of type IV collagen exposure as a molecular imaging target for early detection of thoracic aortic dissection. Theranostics.

[18] Abello J, Nguyen TDT, Marasini R, Aryal S, Weiss ML (2019). Biodistribution of gadolinium- and near infrared-labeled human umbilical cord mesenchymal stromal cell-derived exosomes in tumor bearing mice. Theranostics.

[19] Guo BJ, Yang ZL, Zhang LJ (2018). Gadolinium Deposition in Brain: Current Scientific Evidence and Future Perspectives. Front Mol Neurosci.

[20] McDonald RJ, McDonald JS, Kallmes DF, Jentoft ME, Paolini MA, Murray DL (2017). Gadolinium Deposition in Human Brain Tissues after Contrast-enhanced MR Imaging in Adult Patients without Intracranial Abnormalities. Radiology.

[21] Zhou Z, Lu ZR (2013). Gadolinium-based contrast agents for magnetic resonance cancer imaging. Wiley Interdiscip Rev Nanomed Nanobiotechnol.

[22] Vasan N, Baselga J, Hyman DM (2019). A view on drug resistance in cancer. Nature.

[23] Kim J, Do EJ, Moinova H, Bae SM, Kang JY, Hong SM (2017). Molecular Imaging of Colorectal Tumors by Targeting Colon Cancer Secreted Protein-2 (CCSP-2). Neoplasia.

[24] Zhou Z, Yang L, Gao J, Chen X (2019). Structure-Relaxivity Relationships of Magnetic Nanoparticles for Magnetic Resonance Imaging. Adv Mater.

[25] Zhao H, Richardson R, Talebloo N, Mukherjee P, Wang P, Moore A (2019). uMUC1-Targeting Magnetic Resonance Imaging of Therapeutic Response in an Orthotropic Mouse Model of Colon Cancer. Mol Imaging Biol.

[26] Shuhendler AJ, Ye D, Brewer KD, Bazalova-Carter M, Lee KH, Kempen P (2015). Molecular Magnetic Resonance Imaging of Tumor Response to Therapy. Sci Rep.

[27] Garcia-Figueiras R, Baleato-Gonzalez S, Padhani AR, Luna-Alcala A, Marhuenda A, Vilanova JC (2018). Advanced Imaging Techniques in Evaluation of Colorectal Cancer. Radiographics.

[28] Melemenidis S, Jefferson A, Ruparelia N, Akhtar AM, Xie J, Allen D (2015). Molecular magnetic resonance imaging of angiogenesis *in vivo* using polyvalent cyclic RGD-iron oxide microparticle conjugates. Theranostics.

[29] Schreurs TJL, Jacobs I, Nicolay K, Prompers JJ, Strijkers GJ (2017). Detection of Treatment Success after Photodynamic Therapy Using Dynamic Contrast-Enhanced Magnetic Resonance Imaging. Theranostics.

[30] Jing L, Liang X, Li X, Lin L, Yang Y, Yue X (2014). Mn-porphyrin conjugated Au nanoshells encapsulating doxorubicin for potential magnetic resonance imaging and light triggered synergistic therapy of cancer. Theranostics.

[31] de Bruijn HS, Mashayekhi V, Schreurs TJL, van Driel P, Strijkers GJ, van Diest PJ (2020). Acute cellular and vascular responses to photodynamic therapy using EGFR-targeted nanobody-photosensitizer conjugates studied with intravital optical imaging and magnetic resonance imaging. Theranostics.

[32] Liu Z, Lin H, Zhao M, Dai C, Zhang S, Peng W (2018). 2D Superparamagnetic Tantalum Carbide Composite MXenes for Efficient Breast-Cancer Theranostics. Theranostics.

[33] Han Z, Lu ZR (2017). Targeting Fibronectin for Cancer Imaging and Therapy. J Mater Chem B.

[34] Pankov R, Yamada KM (2002). Fibronectin at a glance. J Cell Sci.

[35] Lu ZR (2017). Magnetic resonance molecular imaging for non-invasive precision cancer diagnosis. Curr Opin Biomed Eng.

[36] White ES, Baralle FE, Muro AF (2008). New insights into form and function of fibronectin splice variants. J Pathol.

[37] Tavian D, De Petro G, Colombi M, Portolani N, Giulini SM, Gardella R (1994). RT-PCR detection of fibronectin EDA+ and EDB+ mRNA isoforms: molecular markers for hepatocellular carcinoma. Int J Cancer.

[38] Coltrini D, Ronca R, Belleri M, Zardi L, Indraccolo S, Scarlato V (2009). Impact of VEGF-dependent tumour micro-environment on EDB fibronectin expression by subcutaneous human tumour xenografts in nude mice. J Pathol.

[39] Han Z, Li Y, Roelle S, Zhou Z, Liu Y, Sabatelle R (2017). Targeted Contrast Agent Specific to an Oncoprotein in Tumor Microenvironment with the Potential for Detection and Risk Stratification of Prostate Cancer with MRI. Bioconjug Chem.

[40] Han Z, Wu X, Roelle S, Chen C, Schiemann WP, Lu ZR (2017). Targeted gadofullerene for sensitive magnetic resonance imaging and risk-stratification of breast cancer. Nat Commun.

[41] Arnold SA, Loomans HA, Ketova T, Andl CD, Clark PE, Zijlstra A (2016). Urinary oncofetal ED-A fibronectin correlates with poor prognosis in patients with bladder cancer. Clin Exp Metastasis.

[42] Santimaria M, Moscatelli G, Viale GL, Giovannoni L, Neri G, Viti F (2003). Immunoscintigraphic detection of the ED-B domain of fibronectin, a marker of angiogenesis, in patients with cancer. Clin Cancer Res.

[43] Midulla M, Verma R, Pignatelli M, Ritter MA, Courtenay-Luck NS, George AJ (2000). Source of oncofetal ED-B-containing fibronectin: implications of production by both tumor and endothelial cells. Cancer Res.

[44] Han Z, Cheng H, Parvani JG, Zhou Z, Lu ZR (2018). Magnetic resonance molecular imaging of metastatic breast cancer by targeting extradomain-B fibronectin in the tumor microenvironment. Magn Reson Med.

[45] Vaidya A, Wang H, Qian V, Gilmore H, Lu ZR (2020). Overexpression of Extradomain-B Fibronectin is Associated with Invasion of Breast Cancer Cells. Cells.

[46] Sun Y, Kim HS, Park J, Li M, Tian L, Choi Y (2014). MRI of breast tumor initiating cells using the extra domain-B of fibronectin targeting nanoparticles. Theranostics.

[47] Sun Y, Kim HS, Saw PE, Jon S, Moon WK (2015). Targeted Therapy for Breast Cancer Stem Cells by Liposomal Delivery of siRNA against Fibronectin EDB. Adv Healthc Mater.

[48] Ye XX, Zhao YY, Wang Q, Xiao W, Zhao J, Peng YJ (2017). EDB Fibronectin-Specific SPECT Probe (99m)Tc-HYNIC-ZD2 for Breast Cancer Detection. ACS Omega.

[49] Park SE, Shamloo K, Kristedja TA, Darwish S, Bisoffi M, Parang K (2019). EDB-FN Targeted Peptide-Drug Conjugates for Use against Prostate Cancer. Int J Mol Sci.

[50] Han Z, Zhang S, Fujiwara K, Zhang J, Li Y, Liu J (2019). Extradomain-B Fibronectin-Targeted Dextran-Based Chemical Exchange Saturation Transfer Magnetic Resonance Imaging Probe for Detecting Pancreatic Cancer. Bioconjug Chem.

[51] Ayat NR, Qin JC, Cheng H, Roelle S, Gao S, Li Y (2018). Optimization of ZD2 Peptide Targeted Gd(HP-DO3A) for Detection and Risk-Stratification of Prostate Cancer with MRI. ACS Med Chem Lett.

[52] Han Z, Zhou Z, Shi X, Wang J, Wu X, Sun D (2015). EDB Fibronectin Specific Peptide for Prostate Cancer Targeting. Bioconjug Chem.

[53] Ayat NR, Vaidya A, Yeung GA, Buford MN, Hall RC, Qiao PL (2019). Effective MR Molecular Imaging of Triple Negative Breast Cancer With an EDB-Fibronectin-Specific Contrast Agent at Reduced Doses. Front Oncol.

[54] Agarwal E, Chaudhuri A, Leiphrakpam PD, Haferbier KL, Brattain MG, Chowdhury S (2014). Akt inhibitor MK-2206 promotes anti-tumor activity and cell death by modulation of AIF and Ezrin in colorectal cancer. BMC Cancer.

[55] Zhu H, Guo W, Zhang L, Davis JJ, Teraishi F, Wu S (2005). Bcl-XL small interfering RNA suppresses the proliferation of 5-fluorouracil-resistant human colon cancer cells. Mol Cancer Ther.

[56] Smith JA, Stallons LJ, Schnellmann RG (2014). Renal cortical hexokinase and pentose phosphate pathway activation through the EGFR/Akt signaling pathway in endotoxin-induced acute kidney injury. Am J Physiol Renal Physiol.

[57] Tang Z, Li C, Kang B, Gao G, Li C, Zhang Z (2017). GEPIA: a web server for cancer and normal gene expression profiling and interactive analyses. Nucleic Acids Res.

[58] Skarkova V, Kralova V, Vitovcova B, Rudolf E (2019). Selected Aspects of Chemoresistance Mechanisms in Colorectal Carcinoma-A Focus on Epithelial-to-Mesenchymal Transition, Autophagy, and Apoptosis. Cells.

[59] Peluso G, Incollingo P, Calogero A, Tammaro V, Rupealta N, Chiacchio G (2017). Current Tissue Molecular Markers in Colorectal Cancer: A Literature Review. Biomed Res Int.

[60] Inufusa H, Nakamura M, Adachi T, Nakatani Y, Shindo K, Yasutomi M (1995). Localization of oncofetal and normal fibronectin in colorectal cancer. Correlation with histologic grade, liver metastasis, and prognosis. Cancer.

[61] Ahmed D, Eide PW, Eilertsen IA, Danielsen SA, Eknaes M, Hektoen M (2013). Epigenetic and genetic features of 24 colon cancer cell lines. Oncogenesis.

[62] Gu C, Wang X, Long T, Wang X, Zhong Y, Ma Y (2018). FSTL1 interacts with VIM and promotes colorectal cancer metastasis via activating the focal adhesion signalling pathway. Cell Death Dis.

[63] Grinde MT, Hilmarsdottir B, Tunset HM, Henriksen IM, Kim J, Haugen MH (2019). Glutamine to proline conversion is associated with response to glutaminase inhibition in breast cancer. Breast Cancer Res.

[64] Reis LMD, Adamoski D, Ornitz Oliveira Souza R, Rodrigues Ascencao CF, Sousa de Oliveira KR, Correa-da-Silva F (2019). Dual inhibition of glutaminase and carnitine palmitoyltransferase decreases growth and migration of glutaminase inhibition-resistant triple-negative breast cancer cells. J Biol Chem.

[65] El-Emir E, Dearling JL, Huhalov A, Robson MP, Boxer G, Neri D (2007). Characterisation and radioimmunotherapy of L19-SIP, an anti-angiogenic antibody against the extra domain B of fibronectin, in colorectal tumour models. Br J Cancer.

[66] Bukowski K, Kciuk M, Kontek R (2020). Mechanisms of Multidrug Resistance in Cancer Chemotherapy. Int J Mol Sci.

[67] McLaughlin R, Hylton N (2011). MRI in breast cancer therapy monitoring. NMR Biomed.

[68] Zhou X, Shan Z, Yang H, Xu J, Li W, Guo F (2018). RelB plays an oncogenic role and conveys chemo-resistance to DLD-1 colon cancer cells. Cancer Cell Int.

[69] Lord ML, Chettle DR, Grafe JL, Noseworthy MD, McNeill FE (2018). Observed Deposition of Gadolinium in Bone Using a New Noninvasive *in vivo* Biomedical Device: Results of a Small Pilot Feasibility Study. Radiology.

[70] Roberts DR, Lindhorst SM, Welsh CT, Maravilla KR, Herring MN, Braun KA (2016). High Levels of Gadolinium Deposition in the Skin of a Patient With Normal Renal Function. Invest Radiol.

[71] Han Z, Sergeeva O, Roelle S, Cheng H, Gao S, Li Y (2019). Preparation and Evaluation of ZD2 Peptide (64)Cu-DOTA Conjugate as a Positron Emission Tomography Probe for Detection and Characterization of Prostate Cancer. ACS Omega.

[72] Gao S, Qin J, Sergeeva O, Sergeev M, Qiao P, Roelle S (2019). Synthesis and assessment of ZD2-((68)Ga-NOTA) specific to extradomain B fibronectin in tumor microenvironment for PET imaging of pancreatic cancer. Am J Nucl Med Mol Imaging.

[73] Tijink BM, Perk LR, Budde M, Stigter-van Walsum M, Visser GW, Kloet RW (2009). (124)I-L19-SIP for immuno-PET imaging of tumour vasculature and guidance of (131)I-L19-SIP radioimmunotherapy. Eur J Nucl Med Mol Imaging.

[74] Mariani G, Lasku A, Balza E, Gaggero B, Motta C, Di Luca L (1997). Tumor targeting potential of the monoclonal antibody BC-1 against oncofetal fibronectin in nude mice bearing human tumor implants. Cancer.

[75] Jailkhani N, Ingram JR, Rashidian M, Rickelt S, Tian C, Mak H (2019). Noninvasive imaging of tumor progression, metastasis, and fibrosis using a nanobody targeting the extracellular matrix. Proc Natl Acad Sci U S A.

