# Supplementary material for: Noninvasive assessment and therapeutic monitoring of drug-resistant colorectal cancer by MR molecular imaging of extradomain-B fibronectin
Source: Theranostics. 2020 Sep 8;10(24):11127–43. doi: 10.7150/thno.47448 (PMC7532678; doi:10.7150/thno.47448)
Supplement: Supplementary file 1 — Supplementary figures. [file thnov10p11127s1.pdf]

Supplementary Figures

Figure 1

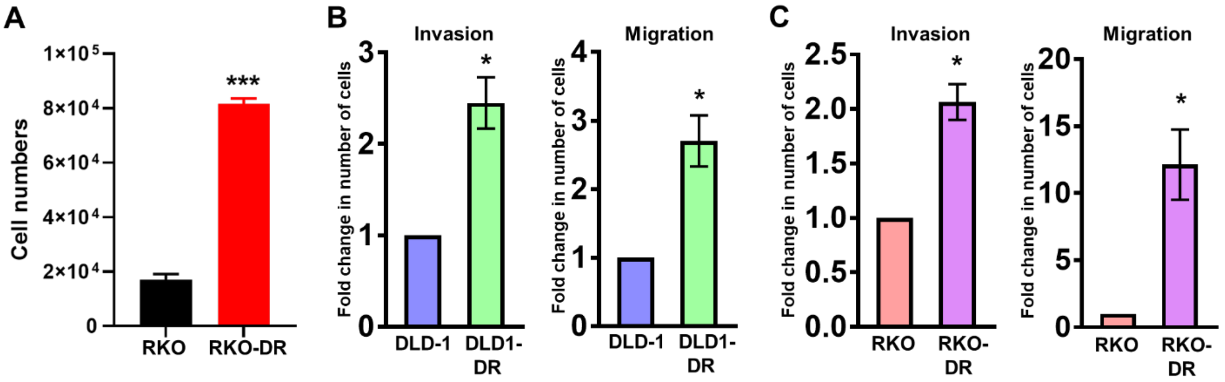

Fig S1. Acquired drug resistance enhances migration and invasion of CRC cells. RKO cells were cultured in 3D Matrigel with 10 µM 5′-FU and 15 µM CB-839 for 4 weeks to select for drug-resistant clones. One of the resistant clones was retested for resistance by another round of treatment on equal number of RKO and RKO-DR cells with both the drugs. After 9 days, cytotoxicity was determined by measuring cell counts. Bars indicate mean ± sem. *p<0.0001 by unpaired *t*-test. Increased invasion through Matrigel layer and migration of the non-resistant and drug-resistant CRC cell lines was measured using transwell assays. Cells were stained using 0.1% crystal violet. After drying, the wells were imaged using Moticam T2 camera, and the cells were counted using FIJI software. Compared to their parent counterparts, the drug-resistant **B.** DLD1-DR and **C.** RKO-DR lines showed significantly higher number of cells invading and migrating through the transwells. Bars indicate mean ± sem. *p<0.05 using unpaired *t*-test.

Figure 2

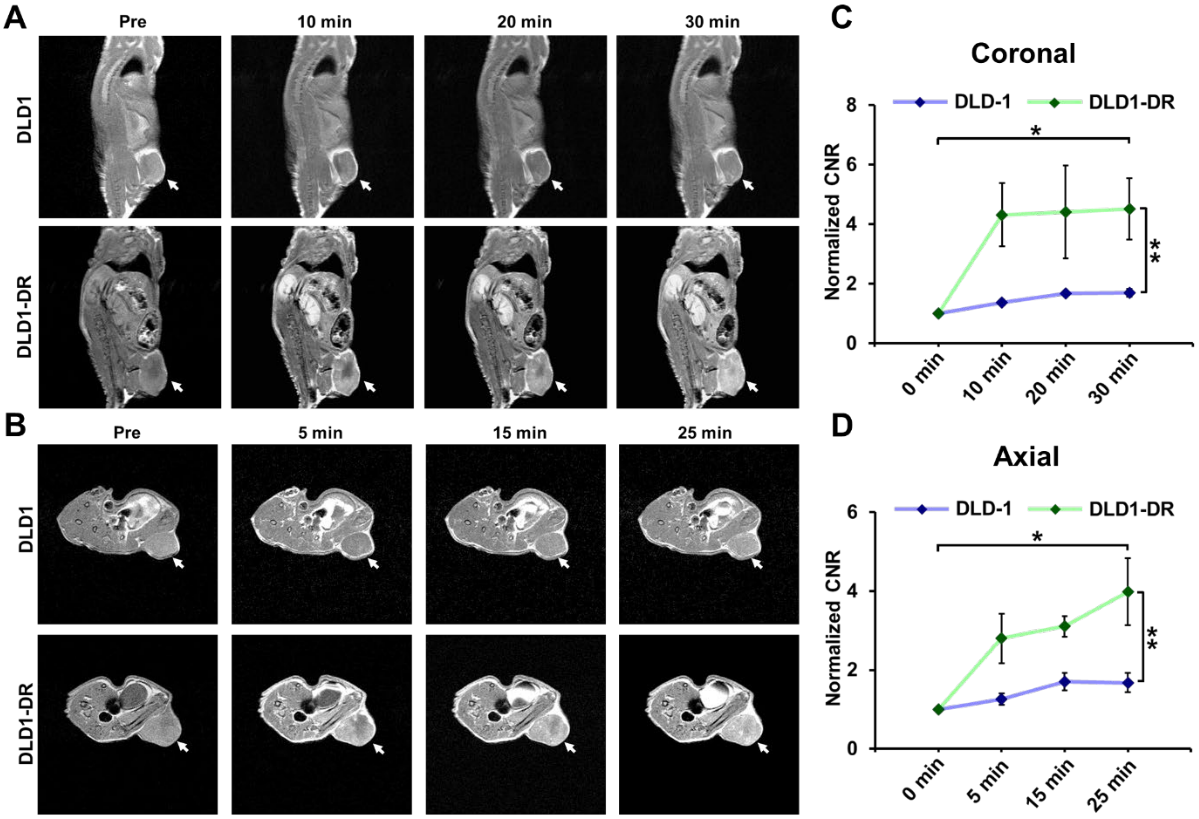

Fig S2. Time course of contrast-enhanced MRMI of EDB-FN using MT218 facilitates non-invasive assessment of drug resistance in DLD-1 tumors. T_1-weighted FSE coronal and axial images were obtained pre- and post-injection (at varying time-points) of 40 µmol/kg dose of MT218 in DLD-1 and DLD1-DR xenograft-bearing athymic nu/nu female mice. Representative **A.** coronal and **B.** axial images show steady increase in signal enhancement for up to 30 min in both DLD-1 and DLD1-DR tumors, with increased enhancement in the latter ones. Significant CNR enhancement is observed in **C.** coronal and **D.** axial images up to 25-30 min in both the tumors compared to pre-contrast, with significantly higher CNR in the DLD1-DR tumors, compared to DLD-1 tumors. (n=2 mice per group, 2 slices per mouse). Dots indicate mean ± sem. *$p<0.05$ (time course) and **$p<0.005$ (DLD-1 vs DLD1-DR) using 2-way ANOVA with Tukey's correction.

Figure 3

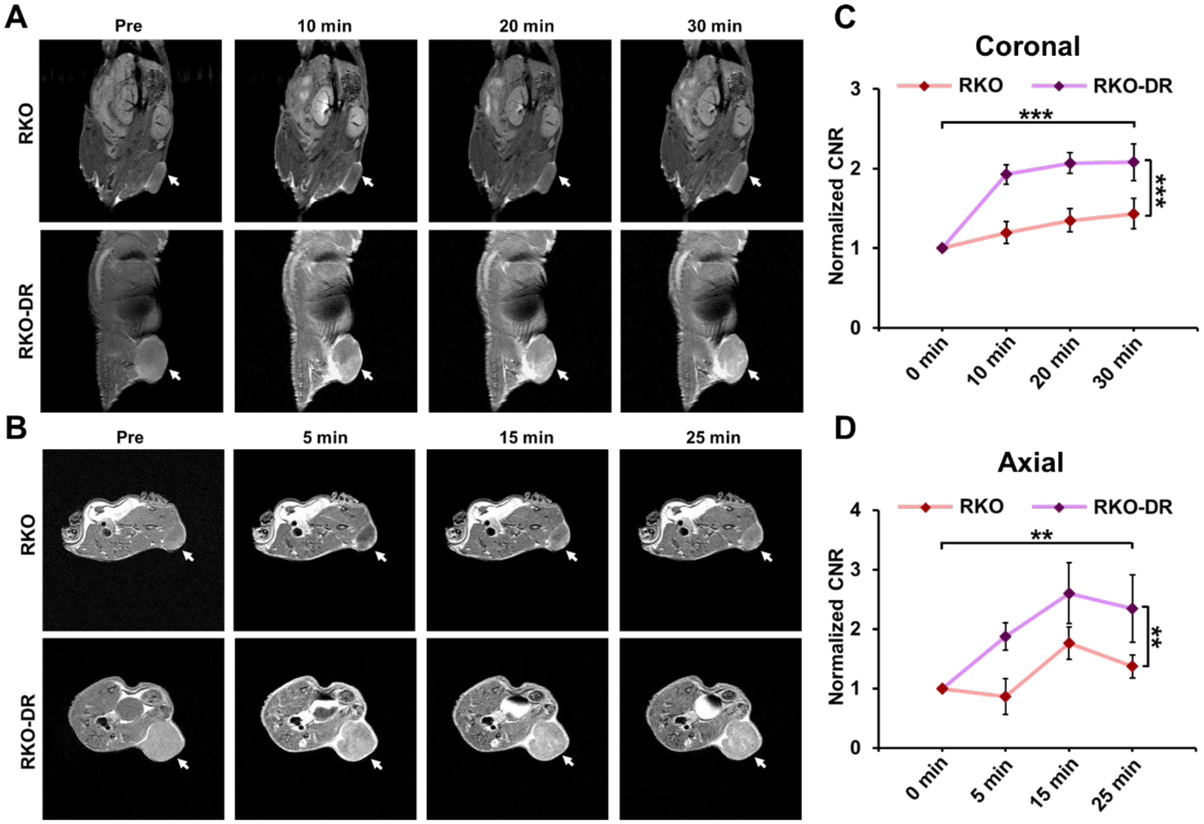

Fig S3. Time course of contrast-enhanced MRMI of EDB-FN using MT218 facilitates non-invasive assessment of drug resistance in RKO tumors. T_1-weighted FSE coronal and axial images were obtained pre- and post-injection (at varying time-points) of 40 µmol/kg dose of MT218 in RKO and RKO-DR xenograft-bearing athymic nu/nu female mice. Representative **A.** coronal and **B.** axial images show steady increase in signal enhancement for up to 30 min in both RKO and RKO-DR tumors, with increased enhancement in the latter ones. Significant CNR enhancement is observed in **C.** coronal and **D.** axial images up to 25-30 min in both the tumors compared to pre-contrast, with significantly higher CNR in the RKO-DR tumors, compared to RKO tumors. (n=2 mice per group, 2 slices per mouse). Dots indicate mean ± sem. ***$p<0.0001$ for time course and RKO vs RKO-DR for C and **$p<0.01$ for time course and RKO vs RKO-DR for D, using 2-way ANOVA with Tukey's correction.

Figure 4

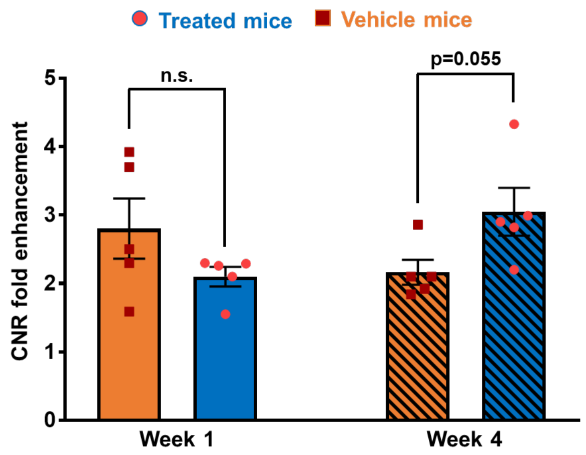

Fig S4. MRMI of EDB-FN with MT218 for non-invasive therapeutic monitoring of mice bearing drug-resistant CRC tumors. CNRs of T_1-weighted 2D spin echo axial images were obtained from MRMI performed at Baseline (week 1) and Endpoint (week 4) with 40 µmol/kg dose MT218 in DLD1-DR-bearing mice treated with DMSO (vehicle group) and MK2206-HCl (treated group). No significant difference observed in the average CNRs between both the groups at week 1. While the week 4 average CNR of the treated group is higher than that of the vehicle group, it is not statistically significant. Bars indicate mean ± sem and dots indicate individual mouse tumor volumes. n=5 mice per group, *p<0.05 using unpaired *t*-test.
